# Predictors of Contraceptive practice in married women of Punjab, Pakistan

**DOI:** 10.12669/pjms.39.3.6575

**Published:** 2023

**Authors:** Wasima Tauseef, Nusrat Jahan Malik, Ikram Ahmad Rana, Fauzia Nusrat Ihsan

**Affiliations:** 1 Dr. Wasima Tauseef, FCPS Department of Obstetrics and Gynaecology Fazle Omar Hospital, Chenab Nagar, Distt. Chiniot, Pakistan; 2 Dr. Nusrat Jahan Malik, MBBS, DOWH (Ireland) Department of Obstetrics and Gynaecology Fazle Omar Hospital, Chenab Nagar, Distt. Chiniot, Pakistan; 3 Dr. Ikram Ahmed Rana, FCPS Department of Cardiology, Tahir Heart Institute Fazle Omar Hospital, Chenab Nagar, Distt. Chiniot, Pakistan; 4 Dr. Fauzia Nusrat Ihsan, MBBS. DOWH (Ireland) Department of Obstetrics and Gynaecology Fazle Omar Hospital, Chenab Nagar, Distt. Chiniot, Pakistan

**Keywords:** Contraception, Family planning, Awareness

## Abstract

**Objective:**

Pakistan is the sixth most populous country in the world. Current contraceptive use in Pakistan is only 26% despite being one of the leading countries in Asia to launch National family planning programs. Major constraint of acceptability among women is the lack of awareness and implementation of contraceptive methods. The objective of this study was to explore the reasons behind this behavior.

**Methods:**

A cross-sectional survey was conducted with non-probability convenient sampling with sample size of 400 married women attending Fazle-Omar Hospital, Chenab Nagar, Punjab, having age between 15 to 60 years from August 2019 to February 2020. To assess the awareness of respondents about contraception, a questionnaire was made after testing its internal consistency. Data was analyzed via SPSS-21; nominal data was expressed as frequencies and percentages, quantitative as mean and standard deviation. Binary logistic regression analysis was carried out to determine predictors for contraception practice. P-value < 0.05 was considered significant.

**Results:**

Our respondents mean age was 30.73±5.9 years. Majority of responders (65%) were educated and (61%) belonged to low socio-economic class. Mean awareness score was 65 ± 26. Out of 400 respondents, 260 (65%) were practicing contraception. Relatives and media were major sources of awareness while clinics and LHVs were contributing less. Condom was the most practiced method of contraception. Low socio-economic class, increase number of kids, responders’ education and awareness score were the predictors of contraception practice.

**Conclusion:**

The education of women and awareness score are independent predictors of contraceptive practice in women. Hence by educating mothers and increasing awareness through various means, practice of contraception can be increased. There is much room to improve the working of family health clinics and LHV.

## INTRODUCTION

The challenges of providing for people’s well-being as well as access to quality health care in countries such as Pakistan can be aggravated by a rapidly growing population. The context for providing family planning is very challenging in Pakistan. The political strife that has intensified over the past two decades and the cultural constraints that limits the empowerment of women have made the effective programs implementation in many parts of Pakistan difficult.^1^ Fertility rate in Pakistan is one of the highest in Asia and rate of contraceptive use is one of the lowest because of which reproductive health is poor and neonatal mortality in Pakistan is high.^2^ Family planning (FP) was acknowledged as one of the vital Millennium Development Goals (MDGs).^3^ It is one of the most health-endorsing and cost-effective activities which prevent maternal deaths approximately 30% and child deaths almost 10%.^4^ It play its role to achieve the MDGs by healthier birth spacing and through reducing pregnancy related mortality and morbidity.^5^ Pakistan was the world 13^th^ largest country in 1950 with a population of 37 million; however; in 2013, Pakistan had become the sixth largest country with a population 191 million.^6^ United Nations has projected Pakistan to move to fifth place with 292 million people in 2050.^1^ Failure to manage the fertility rate results in adverse effects on education, poverty and life expectancy, particularly for mother and child.

The first ever family planning program in Asia was started by Pakistan but paradoxically it is clearly ignored and underutilized.^7^ Literature shows that 59% people avoid contraceptives regardless of its free availability.^8^ If these couples had access to proper information about their use, these unwanted pregnancies would not have happened. Many aspects badly affect use of contraceptive practice like religious and social believes or economic restraints. Due to the problem related with technique tried, the majority of Pakistani women do not practice contraception. Frustration with method, failure of method and experience with side effects are all due to lack of knowledge about how a method works, how its side effects can be managed and to personal preferences which methods are more suited.

To increase the contraceptive methods use in Pakistan, it is important to get the users response. The present study was designed to look into Awareness Score and the choices about contraceptive methods in married women in a local area of Punjab province.

## METHODS

This observational, cross-sectional survey was carried out at Zubaida Bani wing, Fazle-Omar Hospital, Chenab Nagar, Punjab, Pakistan from August 2019 to February 2020 with the approval of Hospital Administration and Ethical Committee (FOH-2415 Dated: 01-08-2019). All married women of age between 15 to 60 years were included through non-probability convenient sampling technique after an informed consent. Minimum sample size to represent 75,000 population of Chenab Nagar, with 5% margin of error and 95% confidence level was 382. It was rounded to 400. Study continued till enrollment of desired 400 respondents sample size.

A questionnaire was prepared comprising of socio-demographic details and assessment of awareness about contraception. The questionnaire comprised of 10 questions initially. The questionnaire was tested for internal consistency on 20 women who met the inclusion criteria. Four questions were eliminated to achieve Cronbach’s alpha reliability coefficient value 0.7 or more suggesting acceptable internal consistency. Four individuals were recruited and trained to assist respondents in filling this questionnaire. After the data collection, the questionnaire was checked before entering the data in SPSS worksheet.

Data was analyzed using SPSS version 21. Continuous variables like age and live births were expressed in mean and standard deviation and nominal data was expressed in frequency and percentage. Ordinal data like socio-economic class (SEC) was expressed as frequency and percentage in each category. Reasons for not practicing contraception were further analyzed in subgroup of women having more than two kids.A binary logistic regression analysis was done to recognize predictors of contraception practice in married women. P-value < 0.05 was considered significant.

### Operational Definitions:

***Educated*:** Education level matriculation or above.

***Socioeconomic Class:***

High = Income Rs.50,000 per month or above.

Middle = Income 20,000-50,000.

Low = Income less than Rs.20,000 per month.

## RESULTS

Four hundred married women of age (15-60 years) fulfilled the criteria of inclusion and gave consent for the study. Our respondents mean age was 30.73 ±5.9 years. Youngest respondent was of age 19 years and eldest was of 56 years. Percentage of educated respondents was 65% and 68% of husbands were educated. Majority of respondents (75%) belonged to urban areas. Majority of respondents were from lower and middle SEC, 61.2% and 29% respectively. Only 9.8% respondents belonged to upper SEC (Table-I).

**Table-I:** Baseline Characteristics.

Age	Mean 30.73 years	±5.9
Urban	299	75%
Rural	101	25%
Respondent (educated)	262	65%
Husband (educated)	274	68%
***Socio-economic class***		
(Low)	245	61%
(Middle)	116	29%
(Upper)	39	10%
No. of pregnancies	3.19	±1.9
No. of live births	2.43	±1.6

Out of 400 respondents, 260 (65%) women were practicing contraception. However, in women who are having more than two kids (n=174), 130 (75%) were practicing contraception, Fear of side effects (39%), want more kids (34%) and husband pressure (11%) were top three reasons for not practicing contraception in women having more than two kids (n=44). There may be more than one reason by each respondent. While looking at whole cohort of non-practicing women (n=140), want more kids (80%), fear of side effects (29%) and unawareness (21%) were top three reasons (Table-II).

**Table II:** Reasons for not practicing contraception .

	All non-practicing women	#	Non-practicing women having > 2 kids	#

	N = 140		N = 44	
Want more kids	80	57.14	15	34.09
Fear of side-effects	29	20.71	17	38.64
Fear of sterility	5	3.57	2	4.55
Husband pressure	6	4.29	5	11.36
Religious belief	1	0.71	1	2.27
Non-availability	4	2.86	4	9.09
Financial reason	7	5	3	6.82
Un-awareness	21	15	4	9.09
Divorced	1	0.71	1	2.27
Widowed	2	1.43	2	4.55
Husband abroad	9	6.43	3	6.82
Medical reason	1	0.71	1	2.27
Want son	1	0.71	1	2.27

**Fig.1:**
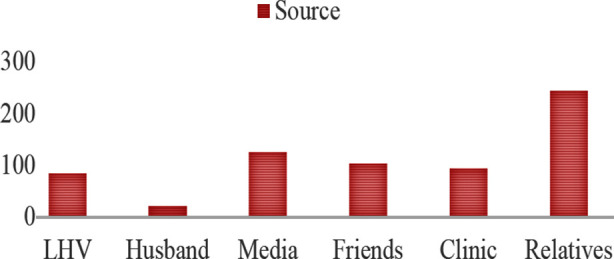
Source of Information.

The sources of information regarding contraceptive methods were mostly relatives (n=244) followed by media (n=125) and friends (n=104). There may be more than one source in each respondent. While husband stayed at the bottom with only 21 respondents. It is important to highlight that Clinics (n=94) and LHV (n=84) were amongst last three sources of information (Fig.1).

**Fig.2:**
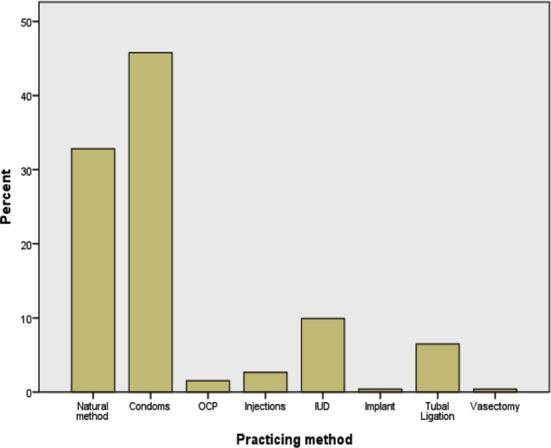
Contraception methods used by respondents.

The more common practiced methods in our study were Condoms (n=120) and Natural methods (n=86) followed by IUCD (n-26) and tubal ligation (n=17) while few were using injectable and oral contraceptives. The implants and vasectomy were utilized minimally as family planning methods (Fig.2).

Mean awareness score was 65 ± 26 and mean time since marriage was 9 ± 6.2 years. Mean awareness score was 70.74 ± 21.34 in subset of women having more than two kids (n=174). This higher awareness score regarding contraception in this subset was translated as higher contraception practice (75% of respondents) as compared to whole cohort of 400 (65%).

Binary logistic regression model was created. Good model fit was supported by Omnibus test (P-value = 0.000) and Hosmer and Lameshow tests (P-value = 0.25). Classification table showed prediction percentage of 74.3%. Binary logistic regression analysis revealed that lower SEC, increase live births, education of responders and increase awareness score were predictors of contraceptive practice in married women. Age of responders, time since marriage, education of husband and urban / rural background did not demonstrate significant P value (Table-III).

**Table-III:** (Binary Logistic Regression analysis)

Variables	B	S.E.	P-value	Odds ratio
Age	-.003	.034	.930	.997
SEC	-.484	.196	.013	.616
Residence	.362	.289	.209	1.437
Responders’ education	.669	.315	.034	1.953
Husbands’ education	.004	.299	.990	1.004
Live births	.337	.118	.004	1.400
Time since marriage	-.006	.040	.889	.994
Awareness Score	.044	.006	.000	1.044
Constant	-2.907	.987	.003	.055

## DISCUSSION

Globally majority of people are well aware of the term ‘family planning’ even if they have never practiced it. Satisfactory maternal health cannot be separated from family planning which demands pertinent contraception use.^2^

In the world, Pakistan is the sixth most populous country with a growth rate of 2.4%.^9^ For a state like Pakistan, which dreads the economic and social implications of uncontrolled growth, addressing population growth is of great concern.^10^

The present study was intended to look into the predictors of contraceptive practice by awareness questionnaire that was made about family planning methods in married women in a local area of province Punjab, Pakistan. In Pakistan, FP begun in the 1960s, also, from year 1964 various methods and strategies were made in this regard. Yet, in the modern age of 21^st^ century, the prevalence rate of contraceptive is recorded as 30%.^11^

In our study 25% of women having more than two kids were not practicing contraception while 75% were practicing it. In one research, in Pakistan 77% women were practicing contraception,^12^ while another study documents that only 26% of women use modern contraceptive devices.^6^ Internationally China has highest contraceptive prevalence which was 89% in 2010^13^ followed by 78.5% in USA.^14^ There has been a slow but sturdy rise in contraception usage with decreasing fertility rate in some countries, like in Germany between 2007 and 2011^15^ and in Spain from 2003 to 2006.^16^

The key point in the adaptation of family planning and making a choice for a particular method is the awareness and knowledge of different contraceptive methods.^17^ Out of the available variety of contraceptive methods, contraceptive method of choice guarantees persistent use and reduces the rate of drop-out.^18^ The approval of client about the method of choice is extremely vital. Clients are well satisfied when they get their choice of method from a variety of contraceptive methods^18^ and choice of contraceptive method becomes limited, if clients do not have the awareness of all contraceptive methods. Methods that are commonly used are female sterilization and condoms which are followed by IUCD.^17^

Few studies have discovered parallel result to our study showing withdrawal^19^ and condoms,^7^,^12^ the most prevailing methods and the reason of condoms being the most popular method often acknowledged in national surveys are ease of use and ease of access.^20^,^21^ Other studies measured oral and injectable hormones effective and commonly used methods.^22^,^23^

In our study, majority of participants identified relatives as a source of information followed by media, friends, clinics and LHVs. Mass media can also play an active role in this regard because media brings the family planning message to the people in their language in a way which is engaging to the listeners.^24^ Method selection is not actually a free choice as most women depends on others guidance to accept a certain contraceptive method. Strong encouragement programs are needed to successfully accomplish family planning goals.^25^

Certain factors such as husband’s education, women’s occupation, women’s mobility, number of living children and husband approval have a huge impact on the family planning methods practice. Another possible factor is the low status of women in the inadequate use of family planning methods in Pakistan.^26^ Among reasons for not practicing contraception, our model revealed fear of side-effects and desire for more children as leading factors. Other studies also show similar results. Leading factors for avoiding contraceptives commonly are difficulty in getting pregnant, the want for more children^27^ and fear of side effects.^28^

### Limitation of study:

For this study, the data collected may not be generalized because participants in the study were volunteers.

## CONCLUSION

Our research proposes that education of women (matriculation or above) and awareness score are independent predictors of contraceptive practice in women. Hence by educating mothers and increasing awareness through various means, practice of contraception can be increased. There is much room to improve the working of family health clinics and LHV as these two were amongst last three out of six sources of awareness that were investigated in this study.

## References

[1] Hardee K, Leahy E (2008). Population, fertility and family planning in Pakistan:a program in stagnation. Pop Action Int.

[2] Aga Khan University, International Advocacy Seminar on Family Planning and Reproductive Health, 12-13, 2013 (Karachi:Department of Community Health Sciences, Aga Khan University, 2013).

[3] Najafi-Sharjabad F, Yahya SZ, Rahman HA, Hanafiah M, Manaf RA (2013). Barriers of modern contraceptive practices among Asian women:a mini literature review. Glob J Health Sci.

[4] Cleland J, Conde-Agudelo APH, Ross J, Tsui A (2012). Contraception and health. Lancet.

[5] Yeakey MP, Muntifering CJ, Ramachandran DV, Myint Y, Creanga AA, Tsui AO (2009). How contraceptive use affects birth intervals:results of a literature review. Studies Fam Plann.

[6] Clifton D, Kaneda T (2013). Family Planning Worldwide 2013 Data Sheet.

[7] Azmat SK, Ali M, Ishaque M, Mustafa G, Hameed W, Khan OF (2015). Assessing predictors of contraceptive use and demand for family planning services in underserved areas of Punjab province in Pakistan:results of a cross-sectional baseline survey. Reprod Health.

[8] Ritter T, Dore A, McGeechan K (2015). Contraceptive knowledge and attitudes among 14–24-year-olds in New South Wales, Australia. Aust N Z J Public Health.

[9] (2017). Government of Pakistan, Provisional summary results of 6^th^ population and housing census 2017 Islamabad:Pakistan bureau of statistics.

[10] Population Council and Bill and Melinda Gate Foundation (2016). Landscape Analysis of the Family Planning Situation in Pakistan. Population Council.

[11] Jamil T, Tanzil S, Ali SS (2018). Influence of Education on Reproductive Health Indicators Among Women in Sindh, Pakistan. Ann Jinnah Sindh Med Uni.

[12] Khan SA, Hafeez H, Akbar R (2015). Emergency contraception:an overview among users. J Ayub Med Coll Abbottabad.

[13] Li J, Temmerman M, Chen Q, Xu J, Hu L, Zhang WH (2013). A review of contraceptive practices among married and unmarried women in China from 1982 to 2010. Eur J Contracept Reprod Health Care.

[14] Callegari LS, Nelson KM, Arterburn DE, Prager SW, Schiff MA, Schwarz EB (2014). Factors associated with lack of effective contraception among obese women in the United States. Contraception.

[15] Ziller M, Rashed AN, Ziller V, Kostev K (2013). The prescribing of contraceptives for adolescents in German gynecologic practices in 2007 and 2011:a retrospective database analysis. J Pediatr Adolesc Gynecol.

[16] Carrasco-Garrido P, Barrera VH, Martin-Lopez R, de Andrés AL, Hernandez JE, Jiménez-García R (2011). Increased use of oral contraceptives in Spain:related factors and time trend, 2003–2006. J Sex Med.

[17] Bibi S, Memon A, Memon Z, Bibi M (2008). Contraceptive knowledge and practices in two districts of Sindh, Pakistan:A hospital based study. J Pak Med Assoc.

[18] Jain AK (1992). Managing quality of care in population programs. In Managing quality of care in population programs.

[19] Sarayloo K, Moghadam ZB, Mansoure JM, Mostafa H, Mohsen S (2015). The impact of an educational program based on BASNEF model on the selection of a contraceptive method in women. Iran J Nurs Midwifery Res.

[20] National Institute of Population Studies (Pakistan), Macro International Institute for Resource Development. Demographic, Health Surveys. Pakistan demographic and health survey. NIPS;2012-2013.

[21] Bibi S, Memon A, Memon Z, Bibi M (2008). Contraceptive knowledge and practices in two districts of Sindh, Pakistan:a hospital base study. J Pak Med Assoc.

[22] Rashed AN, Hsia Y, Wilton L, Ziller M, Kostev K, Tomlin S (2015). Trends and patterns of hormonal contraceptive prescribing for adolescents in primary care in the UK. J Fam Plann Reprod Health Care.

[23] Lutalo T, Gray R, Mathur S, Wawer M, Guwatudde D, Santelli J (2015). Desire for female sterilization among women wishing to limit births in rural Rakai, Uganda. Contraception.

[24] Reshma MS (2015). Awareness in Women Perception for Family Planning:A Case Study of Baliyana Village (Rolitak). Int J Multidiscip Res Dev.

[25] Kols A (2008). Reducing unmet need for family planning:evidence-based strategies and approaches. Outlook.

[26] Ali S, Rozi S, Mahmood MA (2004). Prevalence and factors associated with practice of modern contraceptive methods among currently married women in District Naushahro Feroze. J Pak Med Assoc.

[27] Zuberi SK, Salman SH, Virji RN, Sana S, Kumari S, Zehra N (2015). A hospital-based comparative study of the knowledge, attitudes and practices of family planning among women belonging to different socio-economic status. J Pak Med Assoc.

[28] Amin R (2012). Choice of contraceptive method among females attending family planning center in Hayat Abad Medical Complex, Peshawar. J Pak Med Assoc.

